# Modeling the beating degree of wheat straw biochemical mechanical pulp using multifactorial equations

**DOI:** 10.1371/journal.pone.0339682

**Published:** 2026-01-29

**Authors:** Zihuan Liu, Xiaoli Liang, Xiaoyun Zhang, Ling Li, Liang Yin, Zhenhua Hou, Xiaojie Ma, Yanpeng Xu, Piwu Li, Kaiquan Liu, Ruiming Wang

**Affiliations:** 1 State Key Laboratory of Green Papermaking and Resource Recycling, Qilu University of Technology (Shandong Academy of Sciences), Jinan, Shandong Province, China; 2 Key Laboratory of Shandong Microbial Engineering, College of Bioengineering, Qilu University of Technology (Shandong Academy of Sciences), Jinan, Shandong Province, China; 3 Shandong Century Sunshine Paper Group Co., Ltd., Weifang, Shandong Province, China; 4 Shandong Provincial Key Laboratory of Applied Microbiology, Ecology Institute, Qilu University of Technology (Shandong Academy of Sciences), Jinan, Shandong Province, China; 5 Gansu Engineering Technology Research Center for Microalgae, Hexi University, Zhangye, Gansu Province, China; Koneru Lakshmaiah Education Foundation / Indian and Xidian University, INDIA

## Abstract

In traditional pulp beating processes, the “produce-test-adjust” cycle is commonly employed, often resulting in unnecessary consumption of energy and chemicals. To address this issue, this study integrated single-factor experiments with a Plackett-Burman (PB) design to identify three key parameters—refiner gap, KOH dosage, and enzyme dosage—that significantly influence the beating degree of wheat straw biochemical mechanical pulp, selected from ten potential factors. On this basis, the Box-Behnken Design (BBD) response surface methodology (RSM) was employed to establish a quadratic polynomial predictive model between the beating degree and the aforementioned three factors. For this quadratic polynomial predictive model, the coefficient of determination (R²) is 0.9899, the adjusted R² is 0.9768, and the predicted R² is 0.8723. The adjusted R² is close to R², and the predicted R² is close to the adjusted R² with both values being relatively high, indicating the reliability and practicality of the model. The standard deviation is 0.44, the coefficient of variation is 1.13%, and the signal-to-noise ratio of the model reaches 29.2395, suggesting its strong predictive ability and excellent robustness. Methodologically, this study innovatively applied BBD to the prediction of beating degree. Compared with the traditional Central Composite Design (CCD) model, the proposed model does not require extreme operating conditions, and all 17 experimental points fall within a safe operation range. The establishment of this model provides a predictable and controllable optimization tool for the wheat straw bio-pulping process.

## Introduction

The pulp and paper industry is a crucial pillar of China’s national economy. In 2024, China’s total production of pulp, paper, and paperboard reached 295.93 million tons, marking a year-on-year increase of 1.56%, with market demand continuing to rise [1]. However, the shortage of papermaking raw materials is becoming increasingly prominent. The scarcity of wood fiber resources and the high dependence on imports have become key bottlenecks restricting high-quality industrial development. China possesses abundant non-wood fiber resources, producing approximately 653 million tons of crop straw annually [2]. Efficient conversion of this material into pulping feedstock could alleviate fiber resource scarcity while reducing environmental pollution associated with straw burning [3–6]. Wheat straw, a typical renewable agricultural waste with an average fiber length of 1.32 mm—superior to that of hardwood—and a high cellulose content (30–50%), represents an ideal alternative to wood fiber with broad potential in pulping applications [6–8].

The plant cell wall is a complex three-dimensional network structure consisting of cellulose, hemicellulose (e.g., xylan), and lignin, cross-linked via covalent and non-covalent bonds [9]. However, research on the synergistic application of KOH and composite bio-enzymes in wheat straw pulping remains relatively limited. This study adopts a segmented synergistic approach, involving KOH pretreatment prior to refining and bio-enzyme treatment following refining.

As an alkaline reagent, KOH pretreatment can rapidly dissolve part of the lignin and hemicellulose in the wheat straw fiber intercellular layers, weakening inter-fiber bonding, reducing mechanical energy consumption during refining, and decreasing the probability of excessive fiber cutting [10–12]. This process fully swells and softens the fiber cell wall, expands cell wall porosity, creates conditions for fiber fibrillation during subsequent refining, and results in refined fibers having a larger specific surface area and stronger binding properties [13]. Kataoka et al. studied the effects of KOH concentration and reaction time on jute fiber treatment, reporting that both KOH concentration and reaction time significantly influenced the α-cellulose yield. Spectroscopic analysis confirmed that KOH treatment effectively removed hemicellulose [14]. Pourebrahim et al. investigated the effects of KOH treatment (including KOH concentration, temperature, and soaking time) on date palm fibers, revealing that optimized conditions removed non-cellulosic components (such as hemicellulose and lignin) from the fiber surface, thereby increasing fiber crystallinity and improving fiber-matrix adhesion [15]. Ullah et al. reported that KOH-treated jute/glass and flax/glass hybrid composites successfully eliminated non-cellulosic contaminants, including lignin, hemicellulose, and pectin, enhancing fiber-matrix adhesion and maintaining the dimensional stability of cellulose [16].

Current studies on bio-enzyme treatment primarily focus on the pre-refining stage. Zhang et al. demonstrated that enzymatic pretreatment could reduce pulping chemical consumption by 50% and processing time by 83.3% [17]. Jiang et al. applied bio-enzymes for straw pretreatment and found that appropriate pretreatment time could effectively depolymerize straw fibers and improve fiber bundle properties [18]. In contrast, this study applies bio-enzyme treatment after refining. The addition of composite bio-enzymes (cellulase, pectinase, and xylanase) after refining addresses residual issues post-refining: pectinase removes remaining interlayer pectin and promotes complete fiber dissociation, improving fiber uniformity; xylanase degrades residual hemicellulose on the fiber surface, reducing interference with fiber bonding; and cellulase mildly hydrolyzes the amorphous regions on the fiber surface, promoting fiber fibrillation and enhancing hydrogen bonding between fibers [19–22]. The combined use of these three enzymes, acting simultaneously on the cell wall structure, produces a significant synergistic effect for more efficient degradation.

The core of the pulping process is fiber separation and modification. The beating degree, as a key indicator measuring pulp fiber morphology and binding properties, directly determines the strength, formation, and other essential qualities of the subsequent paper products. In traditional wheat straw pulping, beating degree control relies on the “production-testing-adjustment” cycle, leading to high energy consumption and waste of chemical reagents. In pulping process optimization and performance prediction, statistical modeling methods have gradually replaced traditional single-factor optimization. Mekala et al. developed a statistical regression model for the bagasse kraft pulping process. Using temperature, NaOH charge, and liquor-to-solid ratio as design variables, and pulp yield and Kappa number as response variables, they designed a Central Composite Design (CCD) model. The identified optimal conditions were effective alkali concentration = 4%, liquor-to-solid ratio (V/W) = 25:1, temperature = 160 °C, pulp yield = 46.313%. For Kappa number analysis, the optimal conditions were effective alkali concentration = 3.0%, liquor-to-solid ratio (V/W) = 20:1, temperature = 170 °C, Kappa number = 5.75 [23]. Evelyn et al., using RSM combined with CCD, determined the optimal process conditions for producing kraft pulp from plantain stem. The independent variables included the mass ratio of sodium hydroxide to sodium sulfide, temperature, and time, with pulp yield as the dependent variable. The obtained optimal parameters were temperature 110.50 °C, time 146.88 min, NaOH:Na_2_S ratio 3.372, achieving a pulp yield of 55.064 wt% [24]. Worku et al. employed RSM to explore and determine the optimal conditions for soda treatment of Abyssinian bamboo stems, improving the pulp production process and Kappa number by optimizing soda concentration, cooking temperature, and cooking time. Under optimal conditions (cooking time 180 min, effective alkali 20%, cooking temperature 165 °C), the maximum pulp yield was 43.48% and the minimum Kappa number was 19.9 [25]. However, these studies exhibit limitations: i) most models focus on pulp yield and Kappa number, with few predictive models for beating degree; ii) experimental designs often use CCD, requiring extreme experimental conditions, limiting industrial practicality; iii) most existing models do not systematically screen key influencing factors.

To address these gaps, in this study, we adopted a combined Plackett-Burman (PB) screening and Box-Behnken design response surface methodology (BBD-RSM) to predict the beating degree in wheat straw biochemical mechanical pulping. First, single-factor experiments and PB screening were conducted to rapidly identify key parameters significantly affecting beating degree from ten factors, including wetting and swelling time, wetting and swelling temperature, cooking solid-liquid ratio, cooking time, cooking temperature, KOH dosage, refiner gap, enzyme reaction time, enzyme reaction temperature and enzyme dosage. Subsequently, BBD-RSM was used to construct a quadratic polynomial prediction model linking beating degree with the identified key parameters. By integrating KOH pretreatment with composite enzyme biocatalysis and statistical modeling, this study achieves beating degree prediction, filling the gaps in response objects and experimental design of existing models and providing theoretical support and technical tools for the green and efficient optimization of wheat straw pulping processes. These findings hold significant value for promoting the high-value utilization of non-wood fiber resources and advancing the sustainable development of the paper industry.

Accordingly, the overall aim of this study was to systematically identify the dominant variables affecting the beating degree of wheat straw biochemical mechanical pulp and to establish a predictive model capable of guiding process optimization. Through PB screening and BBD-RSM modelling, this aim was successfully achieved, resulting in a statistically sound and industrially applicable model for controlling beating degree in wheat straw pulping.

## Materials and methods

### Materials

Wheat straw was obtained from Weifang, Shandong, China. After harvesting, wheat straw was subjected to node removal and peeling, cut into small segments (approximately 2 cm in length), washed with water to remove sediment and other non-fibrous impurities, air-dried naturally, and then stored for subsequent use.

Wheat straw is a byproduct of conventional wheat cultivation. In accordance with the *Regulations of the People’s Republic of China on the Protection of Wild Plants* and local agricultural management policies, it is classified as agricultural waste rather than a protected biological resource or regulated natural material. The samples were purchased from local farmers and agricultural product traders through legitimate commercial transactions. The process of sample acquisition did not involve protected natural areas, ecological reserves, or land under special administrative control.

Cellulase, pectinase and xylanase were purchased from Novozymes China Biotechnology Co., Ltd. The cellulase was Viscozyme L with an enzymatic activity of 7500 U/mL (Units/mL); the pectinase was Pectinex XXL with an enzymatic activity of 14000 U/mL; the xylanase was Shearzyme 500 L with an enzymatic activity of 6300 U/mL.

KOH (analytical grade) was purchased from Sinopharm Chemical Reagent Co., Ltd.

## Methods

### Wheat straw biochemical mechanical pulping procedure

Wheat straw (100 g) was mixed with hot water (400 mL) at 35–60 °C for 5–30 min, followed by double refining using a high-intensity continuous disk mill (No. 2500-II, KRK, Japan) with a refiner plate gap of 1 mm. After defibration, the wheat straw was placed in a digester, followed by the addition of 4.2–7.7 g KOH (the KOH dosage was 4.2–7.7% (w/w) relative to the dry weight of wheat straw), the enzymes (cellulase, xylanase, and pectinase) were added at a 1:1:1 activity ratio. Water was added to achieve a solid-to-liquid ratio ranging from 1:2 to 1:10. The system was heated and thoroughly mixed, after which it was placed in a digester (MJ3780D, Deqiang Purification Technology (Shandong) Co., Ltd) at 70–120 °C for 10–60 min. The material was refined in two stages using a high-consistency disk refiner with plate gaps of 0.5 and 0.08–0.5 mm. The ground pulp was soaked in warm water at 60 °C for 10 min. After cooling to room temperature, the pH of the slurry was adjusted to 5.5 using a 1 M H_3_PO_4_ solution, followed by the addition of bio-enzymes at a dosage of 37–222 U (Units). The slurry was placed in a water bath (HH-601A, Changzhou Yinereng Experimental Instrumentation Factory, China) at 35–85 °C for 0–10 h.

The bio-enzyme-treated pulp was screened with a sieving machine (ZQS5, Northwest Institute of Light Industry Machinery Factory, China) to remove screen rejects and obtain the accepted pulp, and the beating degree was determined.

### Single factor experimental design of the beating degree

A single factor experimental design was employed to investigate the influence of individual process parameters on beating degree during pulping as follows:

#### 1. Effect of wetting and swelling on the beating degree.

Following the section *Wheat straw biochemical mechanical pulping procedure*, the effect of wetting and swelling on beating degree was investigated under the following conditions: cooking solid-liquid ratio of 1:8, cooking temperature of 100 °C, cooking time of 40 min, KOH dosage of 5.6%, refiner gap of 0.15 mm, enzyme reaction time of 4 h, enzyme reaction temperature of 55 °C, and enzyme dosage of 74 U.

At a wetting and swelling temperature of 100 °C, experiments were conducted using wetting and swelling times between 5 and 30 min at 5-min intervals, and the beating degree was measured for each condition. Subsequently, when the wetting and swelling time was fixed at 10 min, additional experiments were conducted at wetting and swelling temperatures of 35–60 °C at 5 °C intervals, and the beating degree was recorded.

#### 2. Effect of cooking on beating degree.

Following the section *Wheat straw biochemical mechanical pulping procedure*, the effect of cooking on beating degree was investigated under the following conditions: wetting and swelling time of 10 min, wetting and swelling temperature of 55 °C, KOH dosage of 5.6%, refiner gap of 0.15 mm, enzyme reaction time of 4 h, enzyme reaction temperature of 55 °C, and enzyme dosage of 74 U.

At a cooking temperature of 100 °C and a cooking time of 40 min, experiments were conducted with cooking solid-liquid ratios of 1:2, 1:4, 1:6, 1:7, 1:8, and 1:10, and the beating degree was measured for each condition. When the cooking solid-liquid ratio was fixed at 1:8 and the cooking time was 40 min, additional experiments were performed at cooking temperatures of 70–120 °C at 10 °C intervals, and the beating degree was measured. Furthermore, at a cooking solid-liquid ratio of 1:8 and a cooking temperature of 100 °C, cooking times between 10 and 60 min were evaluated at 10 min intervals, and the beating degree was measured for each condition.

#### 3. Effect of KOH dosage on beating degree.

Following the section *Wheat straw biochemical mechanical pulping procedure*, the effect of KOH dosage on beating degree was investigated under the following conditions: wetting and swelling time of 10 min, wetting and swelling temperature of 55 °C, cooking solid-liquid ratio of 1:8, cooking temperature of 100 °C, cooking time of 40 min, refiner gap of 0.15 mm, enzyme reaction time of 4 h, enzyme reaction temperature of 55 °C, and enzyme dosage of 74 U.

Experiments were conducted with KOH dosages of 4.2–7.7 g (corresponding to 4.2–7.7% w/w relative to wheat straw) at 0.7 g intervals, and the beating degree was measured for each dosage.

#### 4. Effect of refiner gap on beating degree.

Following the section *Wheat straw biochemical mechanical pulping procedure*, the effect of refiner gap on beating degree was investigated under the following conditions: wetting and swelling time of 10 min, wetting and swelling temperature of 55 °C, cooking solid-liquid ratio of 1:8, cooking temperature of 100 °C, cooking time of 40 min, KOH dosage of 5.6%, enzyme reaction time of 4 h, enzyme reaction temperature of 55 °C, and enzyme dosage of 74 U. Experiments were performed with refiner gaps of 0.08, 0.15, 0.20, 0.30, 0.40, and 0.50 mm, and the beating degree was measured for each condition.

#### 5. Effect of enzymatic treatment on beating degree.

Following the section *Wheat straw biochemical mechanical pulping procedure*, the effect of enzymatic treatment on beating degree was investigated under the following conditions: wetting and swelling time of 10 min, wetting and swelling temperature of 55 °C, cooking solid-liquid ratio of 1:8, cooking temperature of 100 °C, cooking time of 40 min, KOH dosage of 5.6%, and refiner gap of 0.15 mm.

At an enzyme reaction temperature of 55 °C and dosage of 74 U, enzymatic treatment times ranging from 0 to 10 h were tested at 2 h intervals using a mixture of cellulase, pectinase, and xylanase at a 1:1:1 activity ratio, and the beating degree was measured. When the enzyme reaction time was fixed at 4 h with a dosage of 74 U, experiments were performed at reaction temperatures from 35 to 85 °C at 10 °C intervals; the beating degree was measured for each condition. Finally, when the reaction temperature of the enzymes was 55 °C and the reaction time was 4 h, enzyme dosages ranging from 37 to 222 U were tested at 37 U intervals, and the beating degree was recorded for each dosage.

### Beating degree design based on PB and BBD-RSM

Based on the results of single-factor experiments, the PB design was employed to identify key variables with significant effects on the beating degree. The PB design, first proposed by Roger Plackett and David Burman in 1946, is an experimental design method developed specifically for identifying critical factors influencing a particular process or system [26]. Its principle relies on binary factor levels (i.e., each factor is set at two levels: low and high), with the core objective of identifying the most influential factors through a minimal number of experiments. This provides guidance for subsequent detailed experimental designs and is particularly suitable for preliminary screening when numerous factors are involved, enabling fast identification. In this study, a total of ten potential factors were investigated at two levels, and 12 experimental runs were designed using Design-Expert 13 software (Stat-Ease, USA).

Following the screening of key variables with significant effects on beating degree via the PB design based on single-factor experimental results, BBD was used to establish a predictive model for beating degree and evaluate interactions and quadratic effects among these variables. BBD is specifically designed for response surface optimization involving 3–5 variables, allowing efficient construction of high-precision quadratic regression models with fewer experimental runs compared with full factorial designs. Unlike CCD, all BBD points lie within the safe operating range, avoiding extreme conditions, thus offering greater practicality.

The three most influential factors—refiner gap, KOH dosage, and enzyme dosage—were selected and set at three levels. A total of 17 experimental runs were generated using Design-Expert 13 software (Stat-Ease, USA). After performing the experiments sequentially, the output response value (i.e., beating degree) was input into the software for statistical model fitting. For these three variables, the statistical model is expressed as:


$$\begin{array}{cc} Y= & \;\beta_{0}+\beta_{1}A+\beta_{2}B+\beta_{3}C+\beta_{12}AB+\beta_{13}AC+\beta_{23}BC\\ & +\beta_{11}A^{2}+\beta_{22}B^{2}+\beta_{33}C^{2}+\varepsilon \end{array}$$ (1)

where Y is the beating degree, and A, B, and C represent refining gap, KOH dosage, and enzyme dosage, respectively. Herein, the constant (β_0_), linear coefficients (β_1_, β_2_, and β_3_), interaction coefficients (β_12_, β_13_, and β_23_), and quadratic coefficients (β_11_, β_22_, and β_33_) were obtained from the 17 experimental runs and analyzed using analysis of variance (ANOVA) in Design-Expert 13 software.

### Determination of the beating degree

The beating degree was measured using a beating degree tester (IMT-DJD02, Dongguan International Material Tester Precision Instrument Co., Ltd.). After sieving, the accepted pulp was air-dried, and a 2.00 g sample (oven-dry equivalent) was weighed, diluted in 1000 mL deionized water, and thoroughly stirred with a stirrer to disperse the fibers uniformly. The wire mesh was inspected to ensure it was clean and undamaged. The scales on all components were verified to be legible, and the apparatus was confirmed to have good airtightness. The slurry was then transferred to the Schopper-Riegler water filtration chamber, and the device was started to allow natural filtration. After the water filtration process stabilizes, observe the liquid level of the filtrate in the graduated cylinder. Read the corresponding °SR value directly from the graduated cylinder, perform parallel determination 3 times, and take the average value as the final beating degree result of the sample.

### Statistical analysis

All statistical analyses were performed using Design-Expert 13 software (Stat-Ease, USA). A p-value <0.05 was considered a statistically significant result.

## Results and discussion

### Effect of different single factors on the pulp beating degree

To investigate the relationship between process variables and beating degree, ten factors were evaluated during the papermaking process: wetting and swelling time (min), wetting and swelling temperature (°C), cooking solid-liquid ratio, cooking temperature (°C), cooking time (min), KOH dosage (%), refiner gap (mm), enzyme reaction time (h), enzyme reaction temperature (°C), and enzyme dosage (U).

### Effect of wetting and swelling

**Fig 1:**
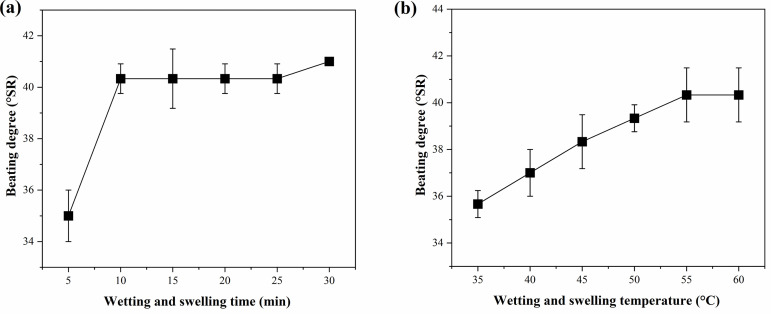
Effect of wetting and swelling on the beating degree of wheat straw pulp. (a) Wetting and swelling time and (b) wetting and swelling temperature. Data represent the mean ± SD of three independent experiments.

The effect of wetting and swelling time on pulp beating degree is shown in Fig 1a. Overall, the beating degree remained stable at approximately 40 °SR as the wetting and swelling time increased, with a pronounced minimum (35 °SR) at 5 min. This phenomenon may be attributed to insufficient initial water absorption by the fibers, preventing them from being fully softened and dissociated. Consequently, the bonding force between fibers during beating is relatively weak, leading to more efficient water filtration performance and a lower beating degree. As wetting and swelling time increased, water gradually penetrated into the amorphous region of cellulose, causing fiber swelling and enhancing fiber flexibility and plasticity. This structural change facilitates fiber fibrillation during beating, generating more fine fibers, increasing the specific surface area and interweaving capacity of fibers, and thereby improving the beating degree.

The effect of wetting and swelling temperature on beating degree is shown in Fig 1b. As the temperature increased from 35 to 55 °C, the beating degree increased from 36 to 40 °SR, exhibiting a clear positive correlation. From a kinetic perspective, higher temperatures accelerate the motion of water molecules and enhance their penetration efficiency into wheat straw fibers. This promotes more rapid water absorption and swelling of the amorphous regions and also promotes the weakening or breaking of hydrogen bonds between fibers, facilitating fiber dissociation into individual fibers or fine components during pulping. Consequently, the water filtration performance decreases, and the beating degree increases significantly. However, excessively high temperatures may cause thermal degradation or over-softening of fibers, impairing their mechanical strength and beating stability. Therefore, an optimal balance between promoting swelling and avoiding thermal damage is required in practical applications.

### Effect of cooking

**Fig 2:**
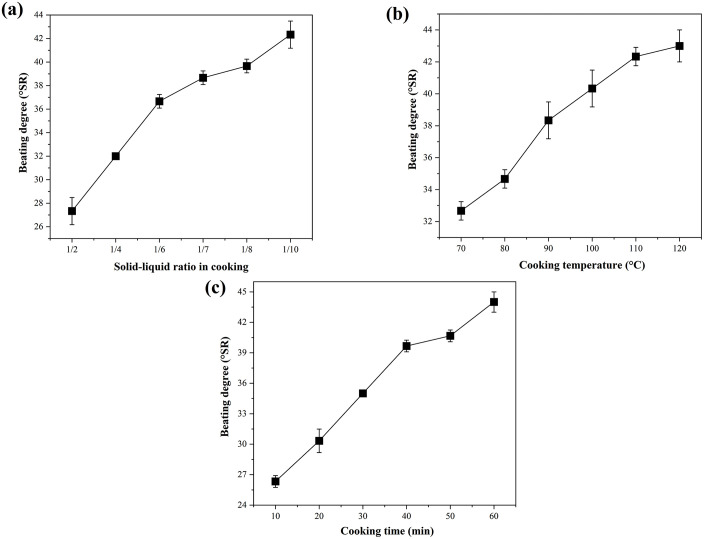
Effect of cooking on the beating degree of wheat straw pulp. (a) Cooking solid-liquid ratio, (b) cooking temperature, and (c) cooking time. Data represent the mean ± SD of three independent experiments.

The cooking solid-liquid ratio refers to the ratio of papermaking raw materials to water during the papermaking cooking process. As shown in Fig 2a, the beating degree of wheat straw pulp increased significantly with increasing liquid-solid ratio, rising from 27 to 42 °SR as the ratio increased from 2:1–10:1. The core mechanism behind this trend is the increase in the liquid phase proportion that improves the accessibility of the KOH chemical liquor, allowing more effective penetration into fiber cell walls. This promotes the dissolution of hemicellulose and lignin, enhances fiber swelling and flexibility, and facilitates fibrillation during subsequent beating. The resulting increase in fiber bonding area ultimately increases the beating degree.

Fig 2b shows that within the experimental temperature range of 70–130 °C, the beating degree of wheat straw pulp increased continuously as cooking temperature increased. The effect of the high-temperature alkaline environment is twofold. First, the increase in temperature accelerates the cleavage of intramolecular chemical bonds (such as ether and ester bonds) in lignin and hemicellulose, weakening inter-fiber bonding and promoting the dissociation of fiber bundles into individual fibers, significantly improving the softening degree of the fibers themselves. Second, higher temperatures reduce fiber rigidity, facilitating morphological changes such as fibrillation and generation of fine fibers during beating. Such changes reduce the mechanical damage to fibers caused by beating and decrease pulp drainability by increasing the specific surface area and interweaving capacity of fibers, thus increasing the beating degree.

As shown in Fig 2c, extending the cooking time from 10 to 60 min resulted in a steady increase in the beating degree of wheat straw pulp. Extending the cooking time allows more thorough interaction between the KOH chemical liquor and the fibers. KOH penetrates deeper into the amorphous regions of the fiber cell walls, fully dissolving lignin and hemicellulose, and further enhancing fiber swelling and flexibility. Furthermore, prolonged cooking promotes the moderate dissolution of fine fibers, which can fill inter-fiber gaps, enhance the bonding force between fibers, and reduce pulp drainage rate. Together, these two factors contribute to a higher beating degree. This trend is consistent with the findings of Abdel-Aal [27], who reported that extending the cooking time (60–120 min) at 170 °C changed fiber structure and reactivity by reducing kappa number and lignin content in buttonwood residues, thereby affecting the beating degree. Such results confirm that the regulatory effect of cooking time on fiber properties and beating performance follows a general and well-established pattern.

### Effect of KOH dosage

**Fig 3:**
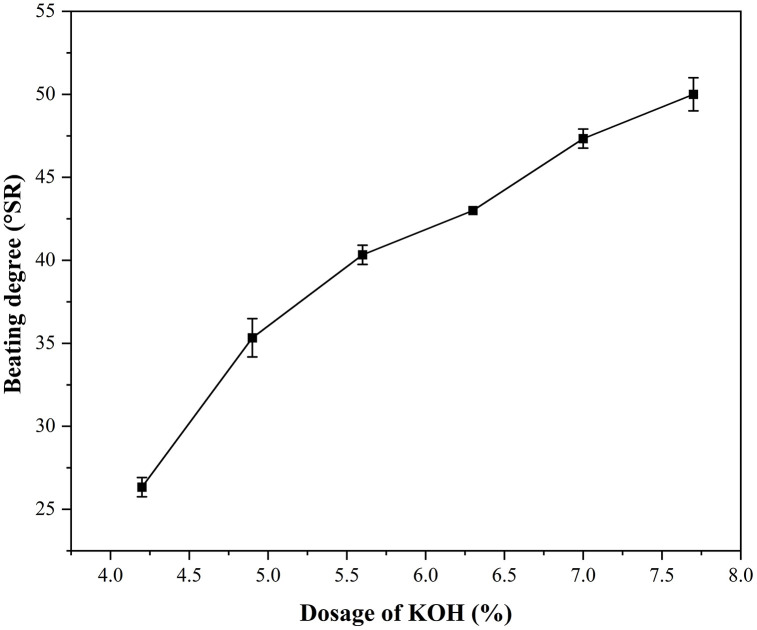
Effect of the KOH dosage on the beating degree of wheat straw pulp. Data represent the mean ± SD of three independent experiments.

KOH dosage is a key determinant of the alkaline cooking efficiency of wheat straw, and its effect on pulp beating degree exhibits a clear dose-dependent relationship. As shown in Fig 3, increasing the KOH dosage from 4.2% to 7.7% raises the pulp beating degree from 25 to 50 °SR. This trend reflects the central role of alkali dosage in the dissolution of fiber components and structural softening. KOH exerts an important influence on the pulp beating degree via its ionization in water, generating K⁺ and OH ⁻ . The OH⁻ ions react with acidic phenolic hydroxyl groups to form water-soluble phenolate salts, which then undergo structural rearrangement and promote the cleavage of the bond linking the oxygen of aryl ether or alkyl ether to the α-carbon of the phenylpropane unit, resulting in the cleavage of the α-ether bond. This leads to subsequent breakdown of β-O-4 (β-aryl ether) linkages, which causes the molecular chains of lignin polymers to be “cleaved” into smaller, soluble fragments [10–12]. As lignin dissolves, more cellulose is exposed and separated. Meanwhile, the high-temperature alkaline environment softens the residual lignin-carbohydrate complexes and hemicellulose, reducing the rigidity of the fibers themselves and enhancing their plasticity. During the subsequent beating process, the fibers are more prone to fibrillation rather than mechanical cutting, increasing specific surface area and enhancing inter-fiber bonding. These changes greatly reduce pulp drainability and ultimately manifest as a substantial increase in beating degree.

### Effect of refiner gap

**Fig 4:**
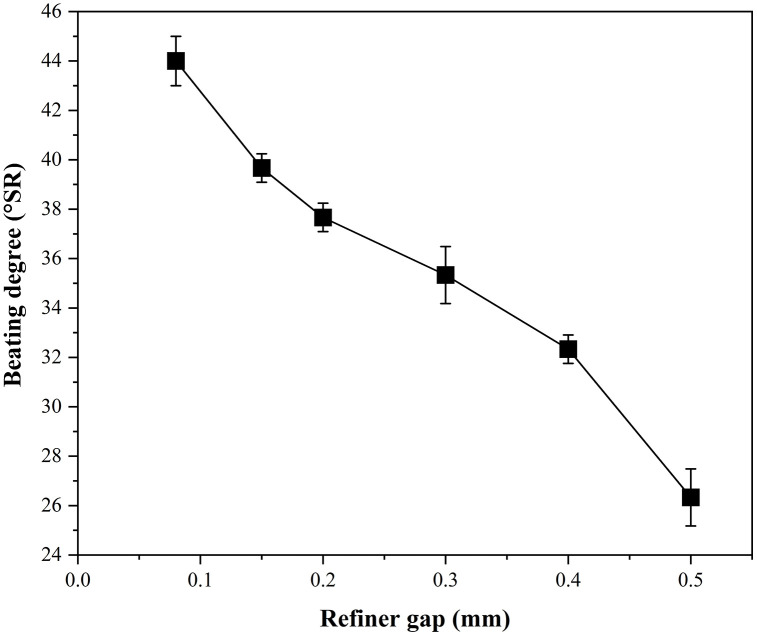
Effect of the refiner gap on the beating degree of wheat straw pulp. Data represent the mean ± SD of three independent experiments.

As shown in Fig 4, increasing the refiner gap results in a lower beating degree. Smaller refiner gaps reduce the average fiber length, increase fibrillation, and produce finer and shorter fibers with stronger inter-fiber bonding. This decreases pulp drainability and increases the beating degree [28].

### Effect of enzymes on the beating degree

**Fig 5:**
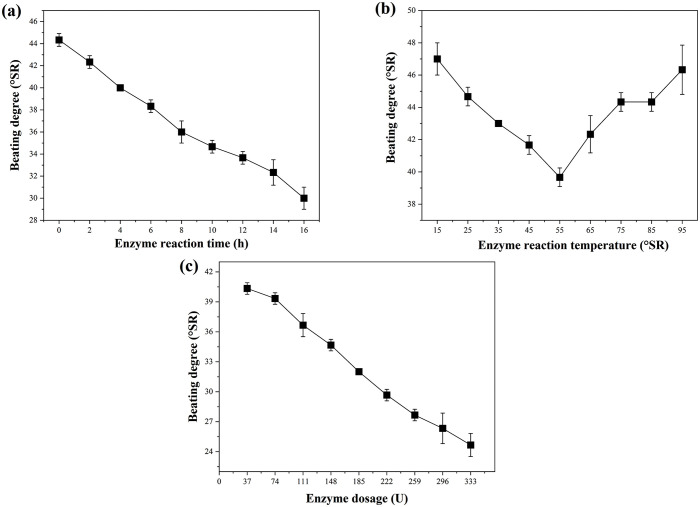
Effect of enzymes on the beating degree of wheat straw pulp. (a) Enzyme reaction time, (b) enzyme reaction temperature, and (c) enzyme dosage on the beating degree. Data represent the mean ± SD of three independent experiments.

As shown in Fig 5a, the beating degree of wheat straw pulp gradually decreases with longer enzymatic reaction time, due to gradual enzymatic deconstruction of the fiber structure. The compound system of cellulase, pectinase, and xylanase forms a “synergistic degradation-accessibility enhancement” action chain. Specifically, pectinase first degrades the pectin components in the pulp, breaking the bonding barrier between fibers and providing more active sites for cellulase and xylanase. Subsequently, xylanase further cleaves the linkage bonds between cellulose and hemicellulose, promoting the dissolution of hemicellulose. Finally, cellulase targets the fine fibers on the fiber surface, causing their fragmentation and dissolution. With the increase in enzymatic reaction time, fiber degradation becomes more sufficient, leading to improved pulp drainability and a decrease in beating degree.

However, a longer enzymatic reaction time is not always beneficial. Beyond an optimal threshold, cellulase may begin degrading the main fiber structure rather than only the surface, causing a significant reduction in fiber length and severe damage to fiber strength. Although the beating degree may remain at a low level (indicating good drainability) under such circumstances, the resulting paper will exhibit markedly reduced tear strength and tensile strength.

The optimal temperature for the composite enzyme solution (composed of cellulase, pectinase, and xylanase) is 55 °C. Below this temperature, increasing heat intensifies the thermal movement of enzyme molecules, improving the binding efficiency between enzymes and substrates and enhancing enzymatic activity. This results in more complete fiber degradation (pectin removal, hemicellulose dissolution, and fine fiber fragmentation), which in turn improves pulp drainability and reduces the beating degree. At 55 °C, the enzymatic activity reaches its peak, achieving optimal degradation and the lowest beating degree. However, when the temperature exceeds 55 °C, the high temperature destroys the spatial conformation of enzyme molecules and gradually inactivates them, reducing their catalytic efficiency. Consequently, fiber degradation is insufficient, leading to an increase in the residual amount of fine fibers, a reduction in pulp drainability, and a rebound in the beating degree (Fig 5b).

As shown in Fig 5c, increasing the enzyme dosage leads to a continuous decrease in beating degree. Higher enzyme concentrations accelerate and deepen fiber modification, reduce fiber rigidity, and promote fiber fibrillation, thereby improving pulp drainability and lowering the beating degree. However, excessive enzyme dosage may cause over-degradation of fibers and damage fiber length, thus negatively affecting tear strength. Furthermore, excessive enzymes may lead to excessive dissolution of cellulose, resulting in a decrease in pulp yield. Therefore, enzyme dosage must be optimized to balance drainability, strength properties, and yield.

### PB multifactorial test design and results

Given the numerous parameters involved in the pulping process, to reduce the number of experiments and experimental costs, the PB experimental design method was employed to efficiently identify the key factors that significantly affect the beating degree. According to the results presented in the previous section, the following ten factors were selected for analysis: wetting and swelling time (min), wetting and swelling temperature (°C), cooking solid-liquid ratio, cooking temperature (°C), cooking time (min), KOH dosage (%), refiner gap (mm), enzyme reaction time (h), enzyme reaction temperature (°C), and enzyme dosage (U). The beating degree of wheat straw pulp was used as the evaluation index and the factors and levels of the PB test were designed using Design-Expert 12 software to screen for significant influencing factors. The factors and levels of the PB experiment are presented in Table 1. The experimental design and results are shown in Table 2. A total of 12 sets of experiments were designed, with three replicates for each set, and the reported values represent the mean of the replicates.

**Table 1:** Factors and levels for the PB design.

Considerations	level	
	−1	1
A – Wetting and swelling time (min)	5	15
B – Wetting and swelling temperature (°C)	50	60
C – Cooking solid-liquid ratio	1:10	1:7
D – Cooking temperature (°C)	90	110
E – Cooking time (min)	30	50
F – KOH dosage (%)	4.9	6.3
G – Refiner gap (mm)	0.08	0.2
H – Enzyme reaction time (h)	2	6
J – Enzyme reaction temperature (°C)	45	65
K – Enzyme dosage (U)	37	111

**Table 2:** PB experimental design.

serial number	1	2	3	4	5	6	7	8	9	10	11	12
Wetting and swelling time (min)	5	5	15	15	15	5	15	5	15	5	15	5
Wetting and swelling temperature (°C)	50	60	60	50	60	50	50	60	50	60	60	50
Cooking solid-liquid ratio	1:7	1:10	1:10	1:10	1:7	1:7	1:7	1:7	1:10	1:10	1:7	1:10
Cooking temperature (°C)	90	110	90	110	90	110	90	110	110	90	110	90
Cooking time (min)	30	30	30	30	30	30	50	50	50	50	50	50
KOH dosage (%)	4.9	4.9	4.9	6.3	6.3	6.3	4.9	4.9	4.9	6.3	6.3	6.3
Refiner gap (mm)	0.08	0.08	0.2	0.2	0.08	0.2	0.2	0.2	0.08	0.2	0.08	0.08
Enzyme reaction time (h)	2	6	2	6	6	2	6	6	2	2	2	6
Enzyme reaction temperature (°C)	45	45	65	45	65	65	45	65	65	45	45	65
Enzyme dosage (U)	37	111	111	37	37	111	111	37	37	37	111	111
Beating degree (°SR)	43	37	37	37	50	34	32	35	48	43	45	48

As shown in Table 3, the P-value of the model is 0.0348 (< 0.05), indicating that the overall model is statistically significant and capable of explaining the variations in beating degree. The factors with significant (P < 0.05) effects are: cooking temperature (D, P = 0.0374), cooking time (E, P = 0.0489), KOH dosage (F, P = 0.0255), refiner gap (G, P = 0.0120), enzyme reaction temperature (J, P = 0.0424), and enzyme dosage (K, P = 0.0277). The F-value reflects the magnitude of the influence. Among these factors, the refiner gap, KOH dosage, and enzyme dosage exhibited the highest F-values (2809, 625, and 529, respectively), indicating that they contribute the most to beating degree variation.

**Table 3:** PB design analysis of variance.

Considerations	Sum of squares	Degrees of freedom	Mean square	F-value	P-value
Model	416.17	10	41.62	499.40	0.0348
A – Wetting and swelling time (min)	6.75	1	6.75	81.00	0.0704
B – Wetting and swelling temperature (°C)	2.08	1	2.08	25.00	0.1257
C – Cooking solid-liquid ratio	10.08	1	10.08	121.00	0.0577
D – Cooking temperature (°C)	24.08	1	24.08	289.00	0.0374
E – Cooking time (min)	14.08	1	14.08	169.00	0.0489
F – KOH dosage (%)	52.08	1	52.08	625.00	0.0255
G – Refiner gap (mm)	234.08	1	234.08	2809.00	0.0120
H – Enzyme reaction time (h)	10.08	1	10.08	121.00	0.0577
J – Enzyme reaction temperature (°C)	18.75	1	18.75	225.00	0.0424
K – Enzyme dosage (U)	44.08	1	44.08	529.00	0.0277
Residual	0.083	1	0.083		
Corrected Total	416.25	11			

**Fig 6:**
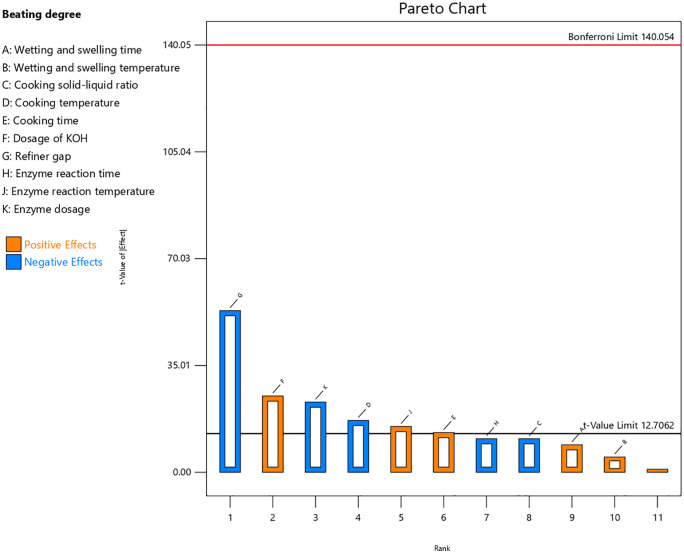
Pareto chart of factors influencing beating degree. Because the PB design is a screening tool that cannot evaluate the interactions between factors, a more comprehensive design is required for subsequent optimization.

As shown in Fig 6, the wetting and swelling time, wetting and swelling temperature, cooking time, KOH dosage, and enzyme reaction temperature have positive coefficients for the beating degree. In contrast, the cooking temperature, refiner gap, cooking solid-liquid ratio, enzyme reaction time, and enzyme dosage have negative coefficients for the beating degree.

### Optimized design and beating degree results

According to the PB results, the refiner gap, KOH dosage, and enzyme dosage were identified as the most influential variables and were therefore selected for further analysis using the BBD-RSM beating degree test (Table 4). A total of 17 sets of experiments were designed, each performed in triplicate, and the experimental results are expressed as the average value (Table 5).

**Table 4:** Factors and levels of the BBD-RSM beating degree test.

considerations	level	
	−1	1
A – Refiner gap (mm)	0.08	0.2
B – Dosage of KOH (%)	4.9	6.3
C – Enzyme dosage (U)	37	111

**Table 5:** BBD-RSM beating degree test design.

Serial number	Refiner gap (mm)	KOH dosage (%)	Enzyme dosage (U)	Beating degree (°SR)
1	0.08	5.6	111	41
2	0.08	6.3	74	45
3	0.08	5.6	37	44
4	0.08	4.9	74	40
5	0.14	4.9	111	36
6	0.14	5.6	74	37
7	0.14	5.6	74	38
8	0.14	6.3	74	40
9	0.14	5.6	74	38
10	0.14	5.6	74	38
11	0.14	4.9	37	38
12	0.14	6.3	37	43
13	0.14	5.6	74	38
14	0.2	5.6	111	35
15	0.2	5.6	37	38
16	0.2	6.3	74	39
17	0.2	4.9	74	35

The resulting quadratic model expressed in terms of coding factors was statistically analyzed:


$$\begin{array}{cc} Y= & \;37\mathrm{.75}-2\mathrm{.88}\times A+2\mathrm{.07}\times B-1\mathrm{.50}\times C-0\mathrm{.25}\times AB\\ & -0\mathrm{.4952}\times BC+1\mathrm{.28}\times A^{\text{2}}+0\mathrm{.6613}\times B^{2}+0\mathrm{.5315}\times C^{\text{2}} \end{array}$$ (2)

where Y (°SR) refers to the pulp beating degree, A (mm) is the refiner gap, B (%) is the KOH dosage, and C (U) is the enzyme dosage.

The contour and response surface plots of the two-by-two effects of the refiner gap, KOH dosage, and enzyme dosage on the beating degree are shown in the supporting information (S1–S6 Figs).

As shown in Table 6, the overall model is highly significant (p < 0.0001), indicating strong predictive capability. The p-values of the three main factors (A, B, and C) are all < 0.0001, confirming their strong influence on the beating degree. In contrast, the interaction terms AB (p = 0.2940), AC (p = 1.0000), and BC (p = 0.1421) are all insignificant, suggesting that each factor affects beating degree primarily through its individual effect. The quadratic terms A² (p = 0.0008) and B² (p = 0.0212) are significant, indicating non-linear relationships and the existence of optimal ranges for refiner gap and KOH dosage. The lack-of-fit term is not significant (p = 0.5016), demonstrating that the model adequately fits the experimental data without systematic errors.

**Table 6:** ANOVA results for the BBD-RSM test.

considerations	Sum of squares	Degrees of freedom	Root mean square	F-value	P-value	
Model	132.34	9	14.74	75.86	< 0.0001	significant
A – Refiner gap (mm)	66.12	1	66.12	340.35	< 0.0001	
B – KOH dosage (%)	28.73	1	28.73	147.90	< 0.0001	
C – Enzyme dosage (U)	12.61	1	12.61	64.92	< 0.0001	
AB	0.25	1	0.25	1.29	0.2940	
AC	0.000	1	0.000	0.000	1.0000	
BC	0.5316	1	0.5316	2.74	0.1421	
A^2^	6.15	1	6.15	31.65	0.0008	
B^2^	1.70	1	1.70	8.75	0.0212	
C^2^	0.9419	1	0.9419	4.85	0.0636	
Residual	1.36	7	0.1943			
Lack of Fit	0.56	3	0.1867	0.9333	0.5016	not significant
Pure Error	0.80	4	0.20			
Cor Total	134.00	16				

From the fitting statistics in Table 7, the coefficient of determination R² = 0.9899 and the adjusted R² = 0.9768, indicate that the model can explain more than 97.68% of the variation in beating degree, demonstrating an excellent goodness of fit. The coefficient of variation (CV% = 1.13%) is low, suggesting good repeatability and minimal experimental error. The signal-to-noise ratio (Adeq Precision = 29.2395) is far higher than the threshold value of 4, indicating that the model has strong and reliable prediction capability. The model yields a mean value of 39 and a standard deviation of 0.44, which further confirms the small discrepancy between the predicted and actual values.

**Table 7:** Fit statistics for equation 1.

Type of statistic	value	Type of statistics	value
Std. Dev.	0.44	R^2^	0.9899
Mean	39	Adjusted R^2^	0.9768
CV%	1.13	Predicted R^2^	0.8723
PRESS	17.11	Adeq. Precision	29.2395

Previous studies commonly employed the CCD-RSM method for model optimization, with most response surfaces designed using pulp yield or Kappa number as indicators [23–25]. In this study, the BBD-RSM method was employed for predicting the beating degree. Unlike CCD, BBD avoids extreme factor levels, ensuring safer operating conditions while requiring fewer experimental runs, thereby leading to higher efficiency.

### Model validation

Model validation was performed using two target beating degrees. For a target of 45 °SR, the model predicted the following process conditions: refiner gap of 0.08 mm, KOH dosage of 6.30%, and enzyme dosage of 68 enzyme activity units (U). In accordance with the procedure described in *Wheat straw biochemical mechanical pulping procedure*, wheat straw was first subjected to washing, rinsing, and defibration treatments, followed by treatment with 6.30% KOH. Subsequently, the material was refined at a refiner gap of 0.08 mm, and finally, 68 U of enzyme was added for enzymatic hydrolysis. The resulting pulp samples exhibited a beating degree of 45 °SR, consistent with the predicted value.

Similarly, the model was validated with a target beating degree of 35 °SR. The process conditions predicted by the model were: refiner gap of 0.2 mm, KOH dosage of 5.06%, and enzyme dosage of 81 U. Experiments were conducted under the aforementioned process conditions, and the resulting pulp exhibited a beating degree of 35 °SR, again showing excellent agreement between experimental and predicted values.

## Conclusion

In this study, through single-factor experiments and PB design, we identified three key parameters with significant effects on the beating degree of wheat straw biochemical mechanical pulp: refiner gap (A), KOH dosage (B), and enzyme dosage (C). Based on these variables, a quadratic polynomial predictive model was established between the beating degree (Y) and the aforementioned three factors using BBD-RSM:


$$\begin{array}{cc} Y= & \;37\mathrm{.75}-2\mathrm{.88}\times A+2\mathrm{.07}\times B-1\mathrm{.50}\times C-0\mathrm{.25}\times AB\\ & -0\mathrm{.4952}\times BC+1\mathrm{.28}\times A^{\text{2}}+0\mathrm{.6613}\times B^{\text{2}}+0\mathrm{.5315}\times C^{\text{2}} \end{array}$$ (2)

where Y (°SR) refers to the pulp beating degree, A (mm) is the refiner gap, B (%) is the dosage of KOH, and C (U) is the enzyme dosage.

The model demonstrated superior fitting performance (R² = 0.9899, adjusted R² = 0.9768), low prediction error (standard deviation = 0.44), and a low coefficient of variation (C.V.% = 1.13%), confirming its reliability and repeatability. The high signal-to-noise ratio (29.2395) further demonstrated strong predictive ability and model robustness. This model provides a predictable and controllable optimization tool for the wheat straw bio-pulping process, with the potential to reduce energy consumption and chemical usage, shorten process adjustment time, and promote the high-value utilization of non-wood fiber resources in support of green papermaking technologies.

Methodologically, this study introduces the novel use of BBD for predicting beating degree. Compared with the traditional CCD approach, BBD avoids extreme experimental conditions, with all 17 experimental points being within the safe operation range. Focusing on the “biochemical mechanical pulping” system, this model incorporates bio-enzyme synergy factors, aligning with the requirements of current green pulping processes.

However, certain limitations remain. All experiments were conducted under laboratory conditions, and complex industrial factors such as equipment variability and continuous production may affect model accuracy. As shown in Table 6, the quadratic terms A² (p = 0.0008) and B² (p = 0.0212) are significant, indicating non-linear effects of the refiner gap and KOH dosage on the beating degree, suggesting the need for further investigation into their optimal operating ranges. Future work will expand the experimental scale, optimize the model to enhance its robustness in industrial scenarios, and conduct additional studies to better define the optimal ranges of refiner gap and KOH dosage.

## Supporting information

S1 Fig Contour diagram of the influence of the refiner gap and KOH dosage on the beating degree. (TIF)

S2 Fig Response surface diagram of the influence of the refiner gap and KOH dosage on the beating degree. (TIF)

S3 Fig Contour diagram of the influence of the refiner gap and enzyme dosage on the beating degree. (TIF)

S4 Fig Response surface diagram of the influence of the refiner gap and enzyme dosage on the beating degree. (TIF)

S5 Fig Contour diagram of the influence of KOH dosage and enzyme dosage on the beating degree. (TIF)

S6 Fig Response surface diagram of the influence of KOH dosage and enzyme dosage on the beating degree. (TIF)
